# Effects of aligner activation and power arm length and material on canine displacement and periodontal ligament stress: a finite element analysis

**DOI:** 10.1186/s40510-023-00492-1

**Published:** 2023-11-27

**Authors:** Aysegul Inan, Merve Gonca

**Affiliations:** 1 Trabzon, Turkey; 2 Department of Orthodontics, Faculty of Dentistry, Recep Tayyip Erdoğan University, Rize, Turkey

**Keywords:** Aligners, Biomechanics, Distalization, FEM, Orthodontic space closure

## Abstract

**Background:**

This study aimed to assess the impact of aligner activation and power arm length and material on canine and aligner displacement, von Mises stress in the power arm, and principal stress in the periodontal ligament (PDL) during canine tooth distalization using finite element analysis (FEA). The effects of aligner activation and power arm length were primary outcomes, while the effect of the power arm material was a secondary outcome.

**Methods:**

Aligner activation (0.1 mm or 0.2 mm) was applied without using a power arm in two models. The effects of aligner activation, power arm length (12, 13, or 14 mm) and power arm material (stainless steel [SS] or fiber-reinforced composite [FRC]) on canine distalization were investigated in 12 models by evaluating displacement and stress via ALTAIR OptiStruct analysis.

**Results:**

Greater canine displacement was observed in all models with 0.2 mm than 0.1 mm of aligner activation. When models with the same aligner activation were compared, reduced mesiodistal tipping, increased palatal tipping, and increased extrusion of the canine cusp were observed with increasing power arm length. Moreover, the von Mises stress increased as the power arm length increased. Increasing the aligner activation and power arm length increased the maximum principal stress in the PDL. Power arms of the same length in both materials showed the same results in terms of canine displacement, clear aligner displacement, and maximum principal stress in the PDL. However, under conditions of equal length and aligner activation, the von Mises stress of the SS power arm was higher than that of the FRC power arm.

**Conclusion:**

Using a power arm in canine distalization reduced mesiodistal tipping but increased palatal tipping and extrusion of the canine cusp. Aligner activation and additional force increased tooth movement and principal stress in the canine PDL. FRC power arms exhibited less von Mises stress than SS power arms.

## Introduction

The rise in adult orthodontic patients has prompted more aesthetic and comfortable alternatives to traditional fixed appliances [1–3]. Since the first introduction by Kesling (1946) of clear aligners (CAs) as thermoplastic tooth positioners, the concept using CAs as a means of achieving tooth movement has gained popularity [4]. In the past, this method has been used for simple corrections, such as in cases of relapse or crowding. However, with the advancement of technology, this method is now also used in more complex cases [5].

The use of CAs in parallel tooth movement is still controversial [6]. Moreover, in cases where four premolars have been extracted and maximum anchorage is needed, closing the extraction gap with a CA is one of the most challenging procedures [7, 8]. Other auxiliary equipment (attachments, buttons, power arms, miniscrews, and elastics) could be required to reduce tipping of the canine toward the extraction gap during canine distalization [9].

Many studies have demonstrated the effects of CA thickness and material, as well as the use of designed attachments, in moving the canine in a parallel manner [10, 11]. In traditional fixed orthodontic treatment, the force must be applied at the center of resistance of the tooth to move it in a parallel manner, or a moment must be created in the tooth with a balanced force couple [12]. Moreover, power arms of different lengths can be used to shift the point of force application closer to the center of resistance [13]. To apply the force closer to the center of resistance in CAs, precision cuts, power ridges, and power arms are required [14]. The power arm provides leverage for a force vector applied to its end, thereby creating a force vector closer to the center of resistance of the tooth and allowing parallel tooth movement to be achieved [15].

In the 1950s, stainless steel (SS) was introduced in the practice of orthodontics as a material component of many devices. Although the release of nickel–chromium from this material may cause adverse reactions (allergic dermatitis) in patients, its low cost, high strength, and ease of formation have made it popular [16]. SS wires and brackets are frequently used in patients, and power arms are also usually made of SS [17]. 

Fiber-reinforced composites (FRCs) are used in the engineering, aviation, and rocket industries. Biomedical products are increasingly used in dentistry to reconstruct craniomaxillary defects, fix removable prostheses, fill resin composites, and fix dental posts [18–20]. In addition to its mechanical properties, FRCs bond well to composite resin and are biocompatible [21–23]. FRCs can be used to maintain spaces and enhance bracket adhesion to the surface of teeth and orthodontic retainers [24]. Furthermore, FRCs can be used to splint groups of two or more teeth and facilitate “en masse” movement through sectional mechanics [25] and thus, are promising substitutes for metals in orthodontics [18, 19]. Valittu et al. reported that FRC wires are suitable alternatives to SS wires [21]. Therefore, the mechanical properties of the corresponding power arm structure are good [15].

Finite element analysis (FEA) is a computational technique used to evaluate stress within an element and determine stress and deformation caused by external forces and pressure. This method is advantageous for assessing the mechanical properties of biomaterials and human tissues, which are challenging to measure directly in vivo [26, 27]. Thus, it can provide insight into the clinical applicability of a biomaterial of interest.

In this study, the effect of aligner activation and power arm length and material on canine and aligner displacement, von Mises stress in the power arm, and principal stress in the periodontal ligament (PDL) in canine distalization was evaluated using FEA.

## Methods

In all, 14 models were designed, with 2 models consisting of 14 teeth with the PDL, alveolar bone, and aligner, and 12 models additionally including a power arm and miniscrew (Table 1).

**Table 1:** Aligner activation, length of the power arm, material of the power arm

Model	Aligner activation (mm)	Length of the power arm (mm)	Material of the power arm
Model 1	0.1 mm	–	–
Model 2	0.1 mm	12 mm	SS
Model 3	0.1 mm	12 mm	FCR
Model 4	0.1 mm	13 mm	SS
Model 5	0.1 mm	13 mm	FCR
Model 6	0.1 mm	14 mm	SS
Model 7	0.1 mm	14 mm	FCR
Model 8	0.2 mm	–	–
Model 9	0.2 mm	12 mm	SS
Model 10	0.2 mm	12 mm	FCR
Model 11	0.2 mm	13 mm	SS
Model 12	0.2 mm	13 mm	FCR
Model 13	0.2 mm	14 mm	SS
Model 14	0.2 mm	14 mm	FCR

*SS* Stainless steel; *FCR* Fiber-reinforced composite; *mm* millimeter

The process of preparing the finite element (FE) models included the following steps: (1) developing the geometric representation of the maxillary dentition alongside its periodontal structures (PDL, cortical and trabecular bone), aligner, attachments, and power arm; (2) transforming the geometric models into FE models; (3) integrating the material properties of the tooth and bone structure, PDL, aligner, attachment, and power arm; (4) establishing boundary conditions; (5) configuring loading conditions; and (6) analyzing and interpreting the results obtained from the simulations [28].

Models were prepared by an MSC Industrial Designer and were analyzed by a mechanical engineer with a Bachelor of Science degree.

### Modeling of the maxilla and orthodontic materials

Computed tomography of a cadaver with well-aligned teeth whose first premolars had been extracted—the canine was at an angle of 90° to the maxillary plane—was used to create a three-dimensional (3D) FE rendering of Hounsfield values in 3DSlicer software (BWH, MA, USA) (https://www.slicer.org/) [29] that was converted into a 3D model by segmentation. Furthermore, 3D FE modeling of the alveolar bone, teeth, and PDL (by surrounding the outer surface of the tooth roots with a thickness of 0.25 mm) was performed using ALTAIR Evolve software (ALTAIR, Troy, MI, USA). Aligner thickness, attachments, and miniscrew scales were modeled based on Xu et al.’s study [30].

The aligner thickness was 0.75 mm, and the aligner completely covered the surfaces of the teeth. Vertical rectangular attachments were placed on the buccal surface of each canine (4 mm in height, 2 mm in width, and 1 mm in thickness), premolar and molar (3 mm in height, 2 mm in width, and 1 mm in thickness). A miniscrew was developed using SolidWorks (Dassault Systemes, USA) [31]; the miniscrew had a diameter of 1.5 mm and length of 10 mm and was positioned between the second premolar and first molar [30]. FE models were converted into mathematical models using ALTAIR HyperMesh software (ALTAIR, Troy, MI, USA). Tria mesh sizes within 0.1–0.25 mm (highly sensitive) were used to create the mathematical models in this study. Moreover, the solid mesh structures of the objects were created as tetrahedrons. These models were imported into the ALTAIR OptiStruct analysis program (ALTAIR, Troy, MI, USA). The number of nodes in the mesh structure of the models is shown in Table 2.

**Table 2:** The number of nodes in the mesh structure of the models

Model	Total of nodes
Model 1, 8	231,553
Model 2, 3, 9, 10	244,237
Model 4, 5, 11, 12	246,728
Model 6, 7, 13, 14	247,405

### 3D coordinate system and definition of boundary conditions

In this study, the x-axis was on the transverse plane, with the positive direction toward the mesial side or surface of the tooth; the y-axis was on the sagittal plane, with the positive direction toward the palatal side or surface; and the z-axis was on the vertical plane, with the positive direction toward the alveolar bone. The models were fixed by restricting all degrees of freedom from the nodal points in the upper region of the cortical bone to prevent 3D movement. The boundary condition was applied to all parts of the model, normal to the x-axis and symmetrical with respect to the y–z plane.

### Material definitions and force loading

The linear material properties of the materials, including the elastic modulus and Poisson’s ratio, are shown in Table 3. All materials in the models were considered homogeneous and isotropic, and PDL was considered a linear isotropic material [30, 32–36].

**Table 3:** Material properties used in the finite element model

Material	Elastic modulus (MPa)	Poisson's ratio	References
Cancellous bone	1370	0.3	Xu Nuo [23]
Compact bone	13,700	0.3	Xu Nuo [23]
Carbon fiber-reinforced composite	129,000	0.33	Vasconcellos [24]
Stainless steel	193,000	0.3	Arifin et al. [25]
Titanium	110,000	0.35	Sarfaraz et al. [26]
Tooth	19,600	0.3	Comba et al. [27]
Composite Attachment	12,500	0.36	Comba et al. [27]
Aligner	528	0.36	Comba et al. [27]
Pdl	0.69	0.45	Amarante et al. [28]

*Mpa* megapascal; *Pdl* periodontal ligament

Nonlinear friction with a coefficient of *µ* = 0.2 was assigned to the aligner attachment and aligner-tooth interfaces [37]. The type of contact at the points of aligner–power arm, tooth–PDL, tooth–attachment, and bone–screw contacts was defined as fixed as the parts moved with complete correlation [38].

**Fig. 1:**
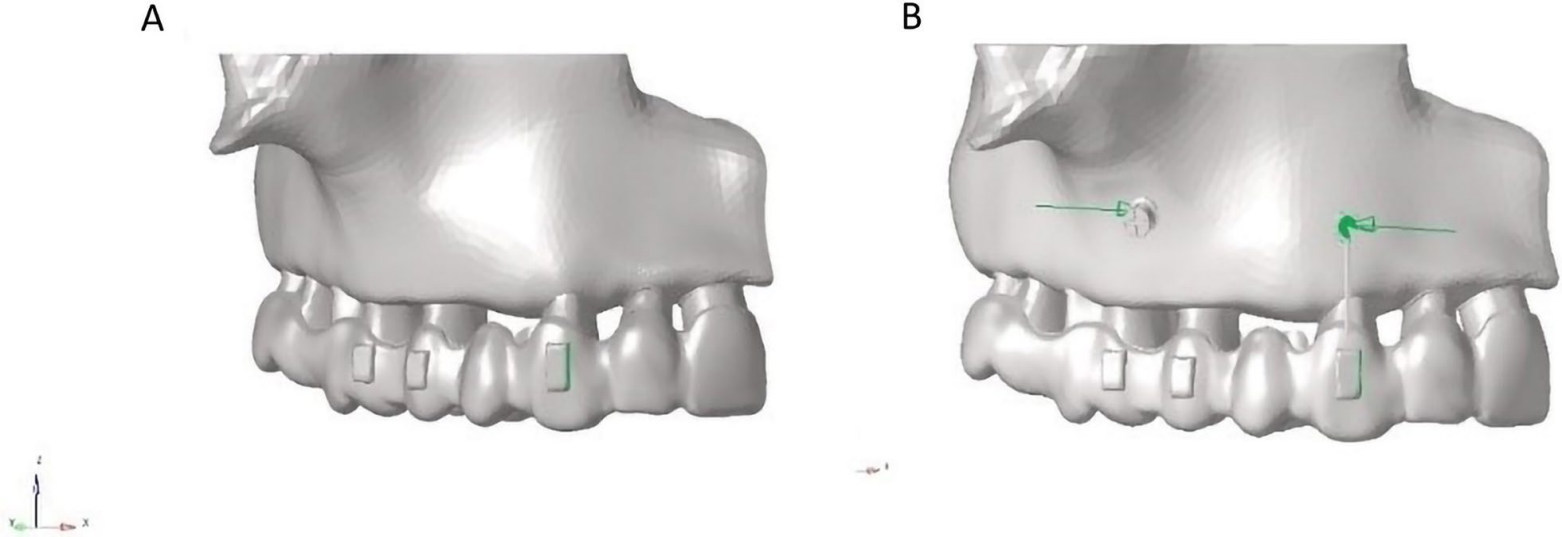
Demonstration of force application. (**A**) Force was applied only to the mesial aspect of the canine attachment with the aligner. (**B**) Force was applied from the power arm to the miniscrew by means of the elastic in addition to the mesial aspect of the canine attachment

Two scenarios of force application were created for canine distalization. In the first, the distal force was applied to the canine using a CA (Fig. 1A). In the second, the distal force was applied to the canine using both a CA and a power arm from a miniscrew (Fig. 1B). The CA activation was 0.1 mm or 0.2 mm. Power arms of different lengths (12, 13, or 14 mm) and of two types of material (SS or FRC) were modeled. In 2 of these models, an aligner activation of 0.1 mm or 0.2 mm was applied with no power arm. An additional 12 models were established according to the combination of 2 aligner activations (0.1 or 0.2 mm), 3 power arm lengths (12, 13, or 14 mm), and 2 power arm materials (SS or FRC); thus, 14 models were created. The effects of aligner activation and power arm length were primary outcomes of the study, while the effect of the power arm material was the secondary outcome.

The center of resistance of the canine is located at approximately two-fifths of the root length from the alveolar margin, according to Vollmer et al. [39] In this study, the direction of the force vector applied by the 12 mm, 13 mm, and 14 mm power arm was located below, at, and above the center of resistance, respectively.

In our study, a 4-oz elastic force was applied between the power arm and the miniscrew, similar to that described by Colomba et al. [35].

### Statistical analysis

In FE studies, validating the results obtained by the software tools through FE simulation is sufficient, thereby warranting the need for experimental readings. Thus, statistical analysis was not required [40].

## Results

**Fig. 2:**
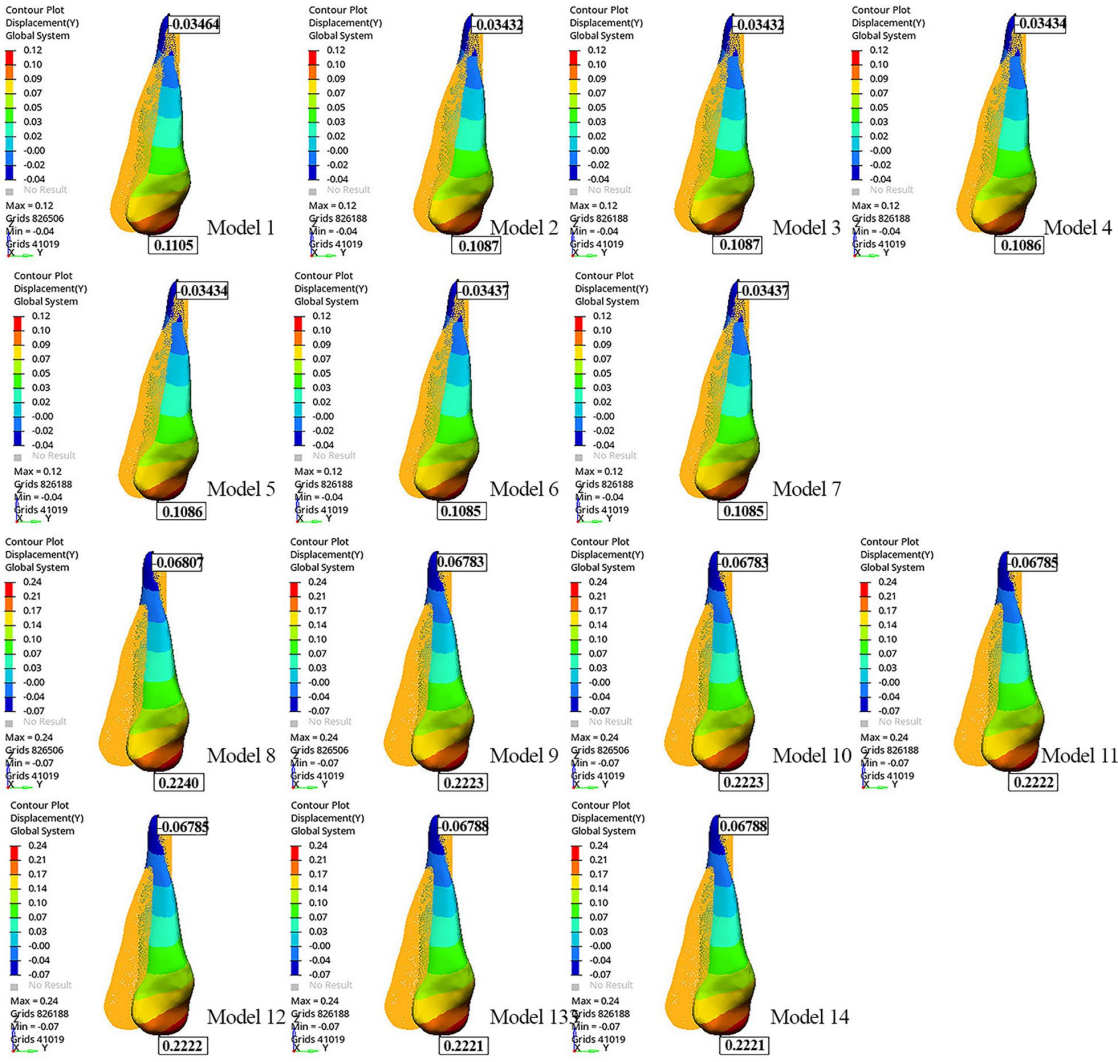
Displacement of the canine along the y-axis

The tip of the canine cusp was displaced distally, while the apex of the canine root was displaced mesially in all models (Table 4). The greatest movement at the apex of the canine root and the tip of the canine cusp was observed in Model 8 (− 0.06807 mm and 0.2240 mm, respectively) (Fig. 2; Table 4). As the length of the power arm increased, the mesiodistal tipping of the canine decreased.

**Table 4:** Displacements of the tip of the canine crown and apex of the root along the x, y, z axis

Models	Displacements of the canine (mm)					
	X		Y		Z	
	Crown	Root	Crown	Root	Crown	Root
Model 1	0.03994	− 0.01579	0.1105	− 0.03464	− 0.01914	0.01922
Model 2	0.04026	− 0.01570	0.1087	− 0.03432	− 0.01839	0.01946
Model 3	0.04026	− 0.01570	0.1087	− 0.03432	− 0.01839	0.01946
Model 4	0.04051	− 0.01580	0.1086	− 0.03434	− 0.01855	0.01932
Model 5	0.04051	− 0.01580	0.1086	− 0.03434	− 0.01855	0.01932
Model 6	0.04078	− 0.01591	0.1085	− 0.03437	− 0.01873	0.01917
Model 7	0.04078	− 0.01591	0.1085	− 0.03437	− 0.01873	0.01917
Model 8	0.07924	− 0.02871	0.2240	− 0.06807	− 0.04045	0.03939
Model 9	0.07959	− 0.02853	0.2223	− 0.06783	− 0.03996	0.03942
Model 10	0.07959	− 0.02853	0.2223	− 0.06783	− 0.03996	0.03942
Model 11	0.07984	− 0.02862	0.2222	− 0.06785	− 0.04013	0.03928
Model 12	0.07984	− 0.02862	0.2222	− 0.06785	− 0.04013	0.03928
Model 13	0.08011	− 0.02873	0.2221	− 0.06788	− 0.04032	0.03913
Model 14	0.08011	− 0.02873	0.2221	− 0.06788	− 0.04032	0.03913

*mm* millimeter

**Fig. 3:**
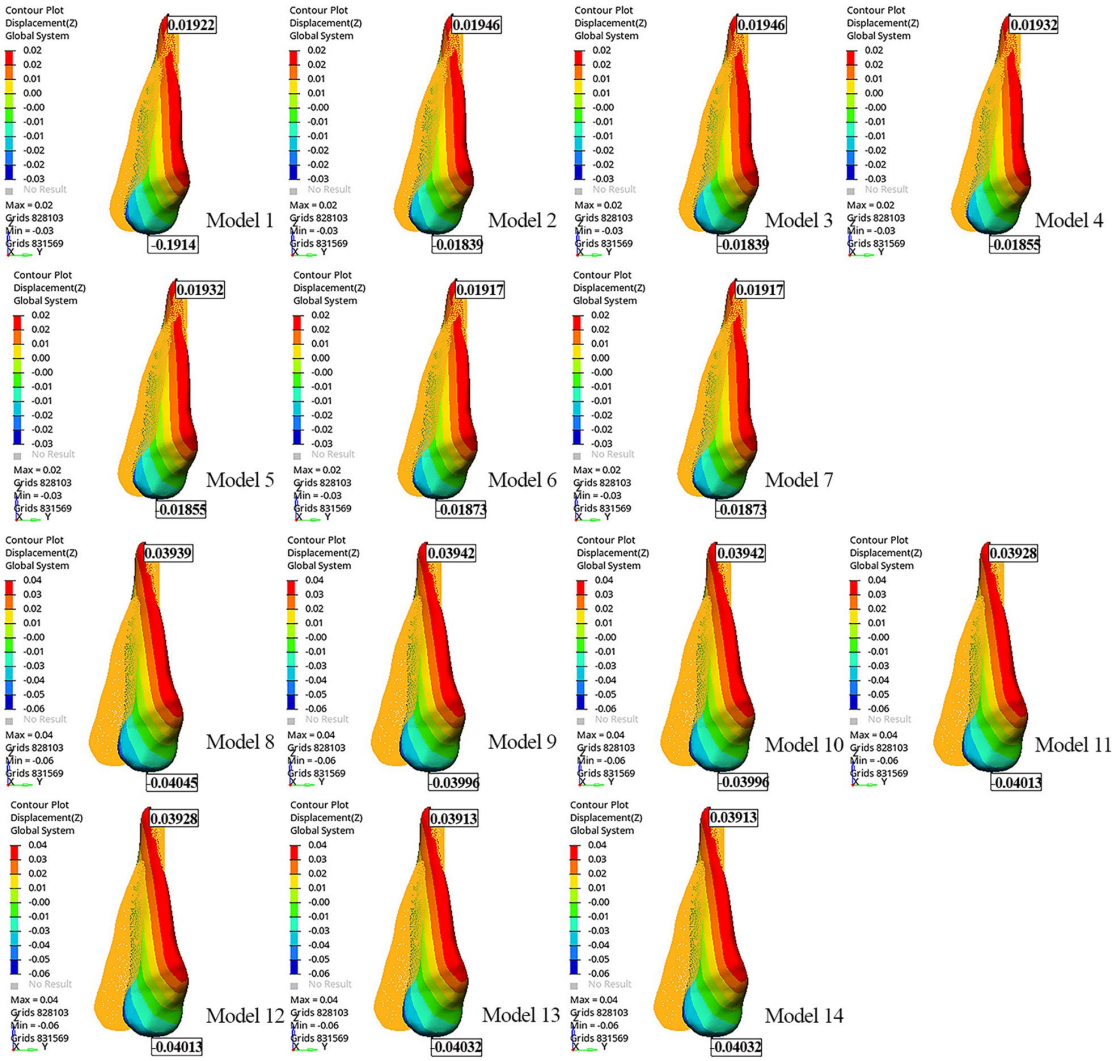
Displacement of the canine along the z-axis

Extrusion was observed at the tip of the canine cusp in all models (Fig. 3). Model 8 showed greater extrusion of the tip of the canine cusp than the other models (− 0.04045 mm). The greatest movement at the apex of the canine root was observed in Models 9 and 10 (0.03942 mm) (Fig. 3; Table 4). When the models with power arms were compared among themselves, the tip of the canine cusp extruded more as the power arm length increased.

**Fig. 4:**
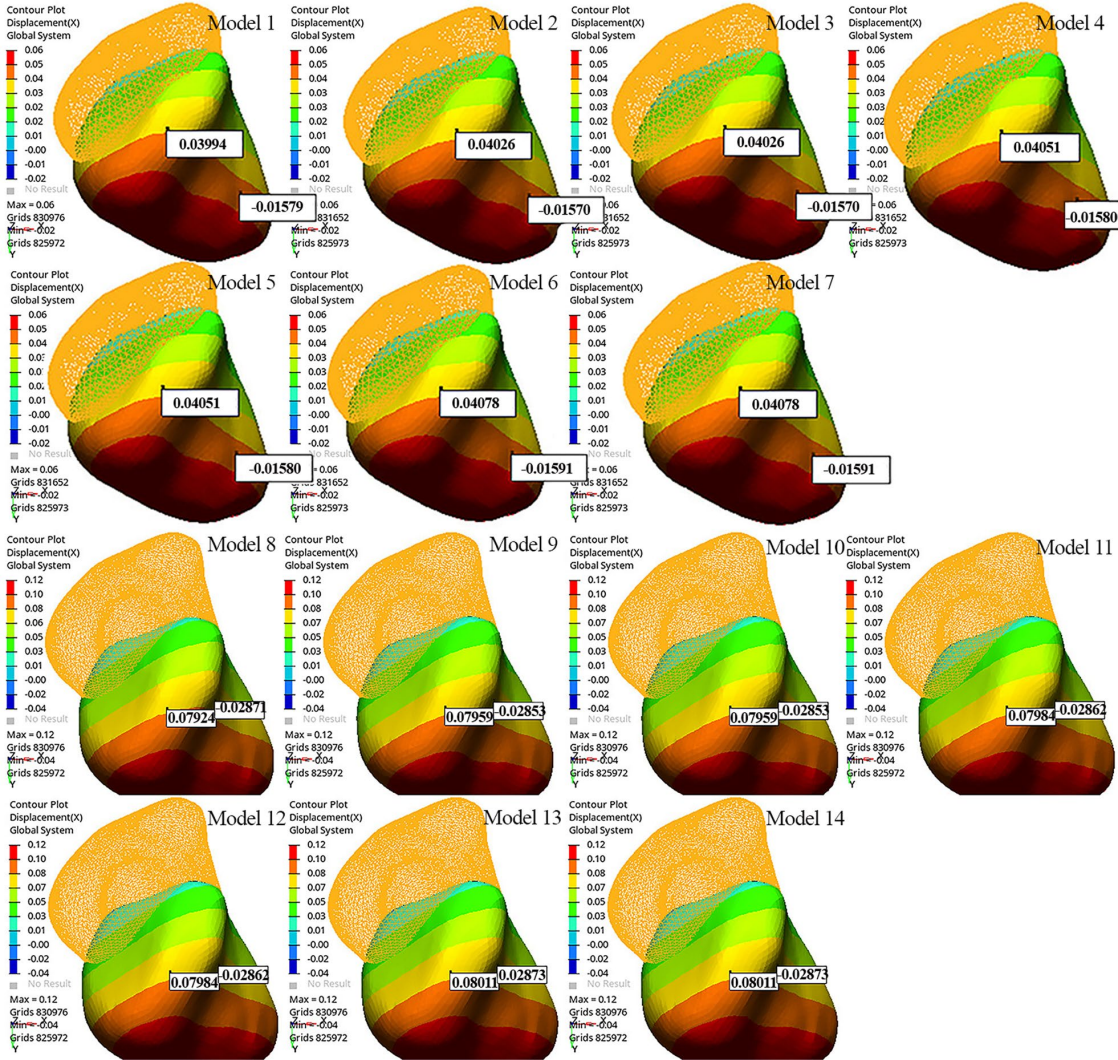
Displacement of the canine along the x-axis

In all models, palatal displacement was observed at the tip of the canine cusp, and buccal displacement was observed at the apex of the canine root. The tip of the canine cusp and apex of the canine root were displaced more in Models 13 and 14 than in the other models (0.08011 mm and − 0.02873 mm, respectively). Distopalatal rotation of the crown of the canine was observed, mostly in the distobuccal region (Fig. 4). When the models with the same aligner activations were compared among themselves, Models 1 and 8 showed the least palatal displacement of the canine crown (Fig. 4; Table 4). As the length of the power arm increased, the palatal displacement of the canine crown and the buccal displacement at the apex of the canine root also increased. Root torque was most preserved in models with a 12-mm power arm, with the least buccal movement of the root (Table 4).

**Fig. 5:**
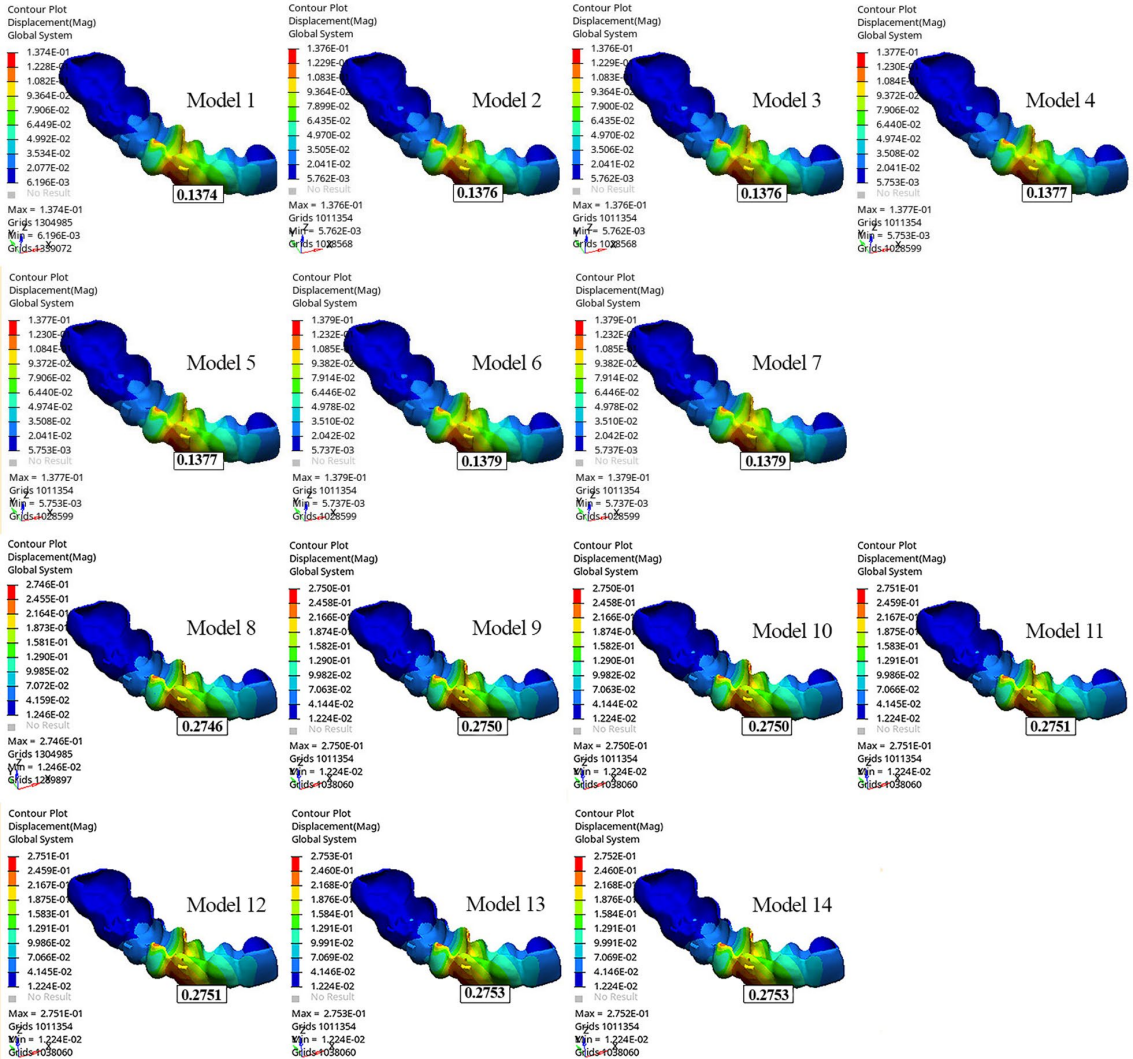
Displacement of the clear aligner

**Fig. 6:**
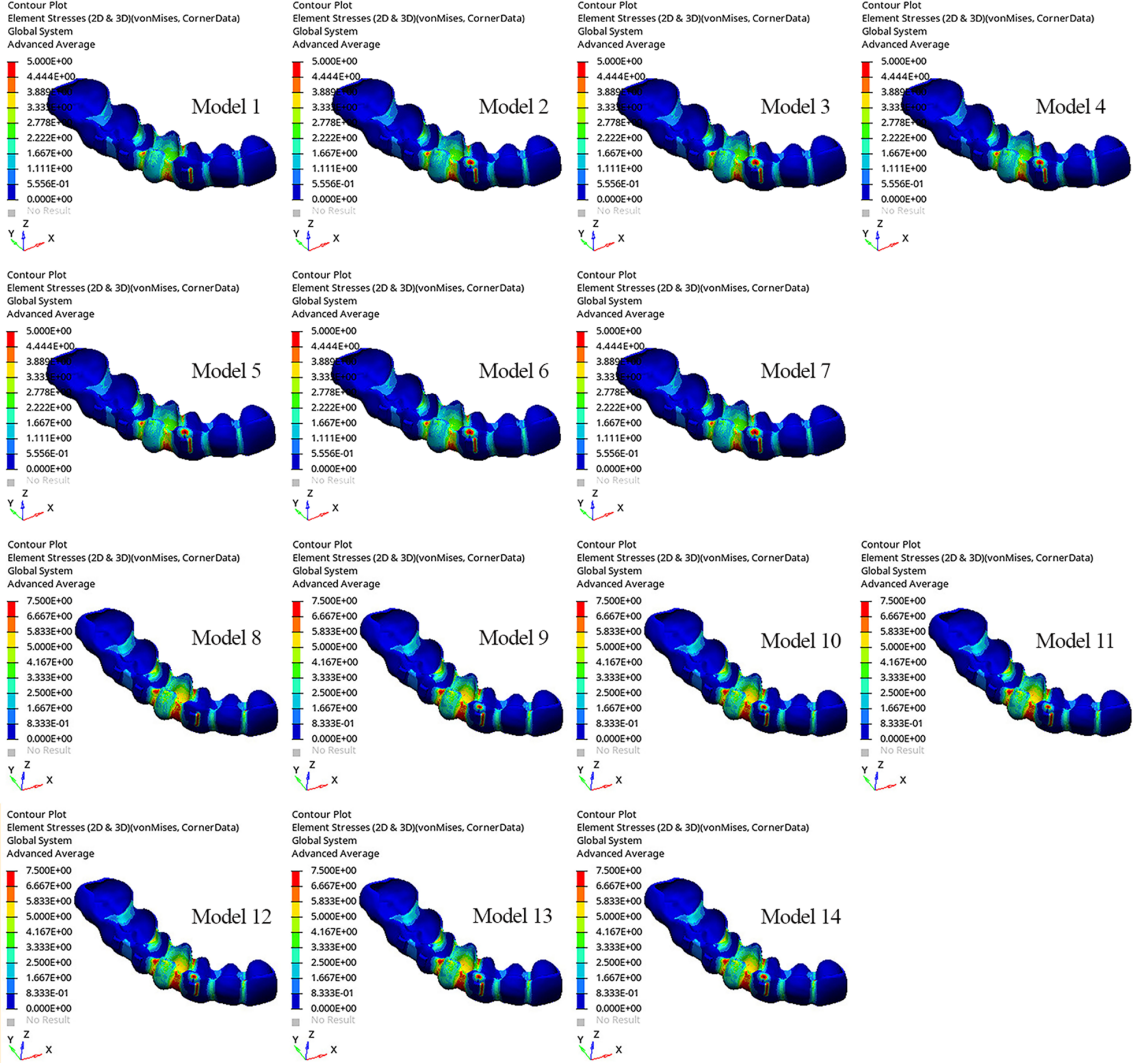
Von Mises stress of the aligner

The greatest displacement of the aligner was demonstrated at the distobuccal region of the canine crown (Fig. 5). Moreover, the greatest displacement occurred in Models 13 and 14 (0.2753 mm), and the least displacement occurred in Model 1 (0.1374 mm) (Fig. 5; Table 5). The greatest von Mises stress was observed on the aligner covering the mesial surface of the extracted first premolar and that of the vertical attachment of the canine. The von Mises stress was greater in all models with 0.2 mm of aligner activation than in those with 0.1 mm of aligner activation. When the models were classified and evaluated according to the aligner activation (0.1 mm or 0.2 mm), the von Mises stress of the aligner covering the buccogingival line of the canine was greater in models with power arms than in models without power arms (Fig. 6).

**Table 5:** Displacement of the clear aligner

Models	Displacement of the clear aligner (mm)
Model 1	0.1374
Model 2	0.1376
Model 3	0.1376
Model 4	0.1377
Model 5	0.1377
Model 6	0.1379
Model 7	0.1379
Model 8	0.2746
Model 9	0.2750
Model 10	0.2750
Model 11	0.2751
Model 12	0.2751
Model 13	0.2753
Model 14	0.2753

*mm* millimeter

**Fig. 7:**
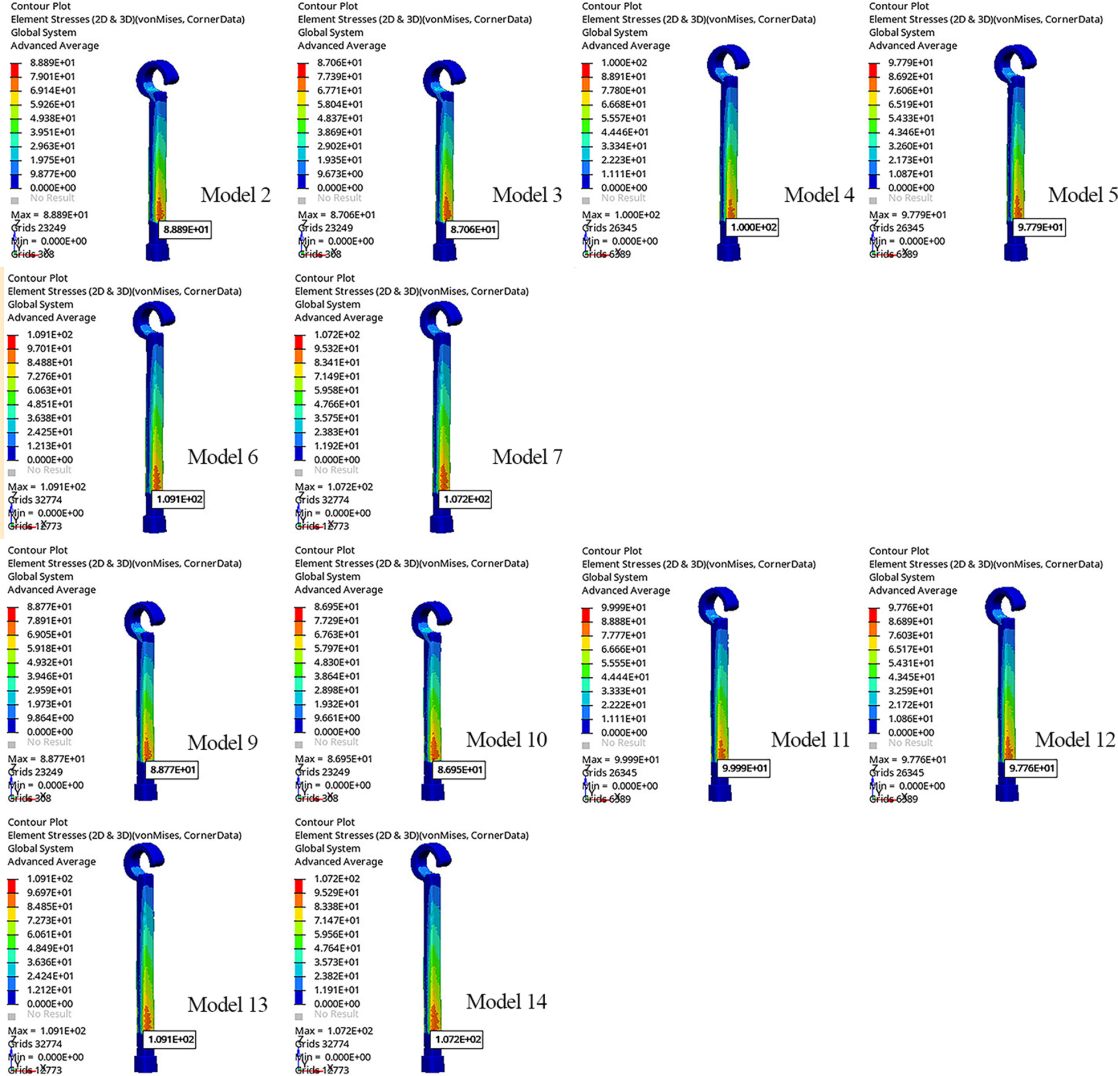
Von Mises stress of the power arm

The von Mises stress in the power arm increased as the length of the power arm increased. The lowest von Mises stress was observed in Model 3, at 87.06 MPa (Fig. 7; Table 6).

**Table 6:** Max Von mises stress values and the location on the power arm

Models	Max. stress (Mpa)	Max. stress location (mm)
Model 1	–	–
Model 2	88.89	2.687
Model 3	87.06	2.691
Model 4	100.00	2.703
Model 5	97.79	2.707
Model 6	109.1	2.702
Model 7	107.2	2.705
Model 8	–	–
Model 9	88.77	2.682
Model 10	86.95	2.687
Model 11	99.99	2.707
Model 12	97.76	2.708
Model 13	109.1	2.705
Model 14	107.2	2.707

*max* maximum; *Mpa* megapascal; *mm* millimeter

**Fig. 8:**
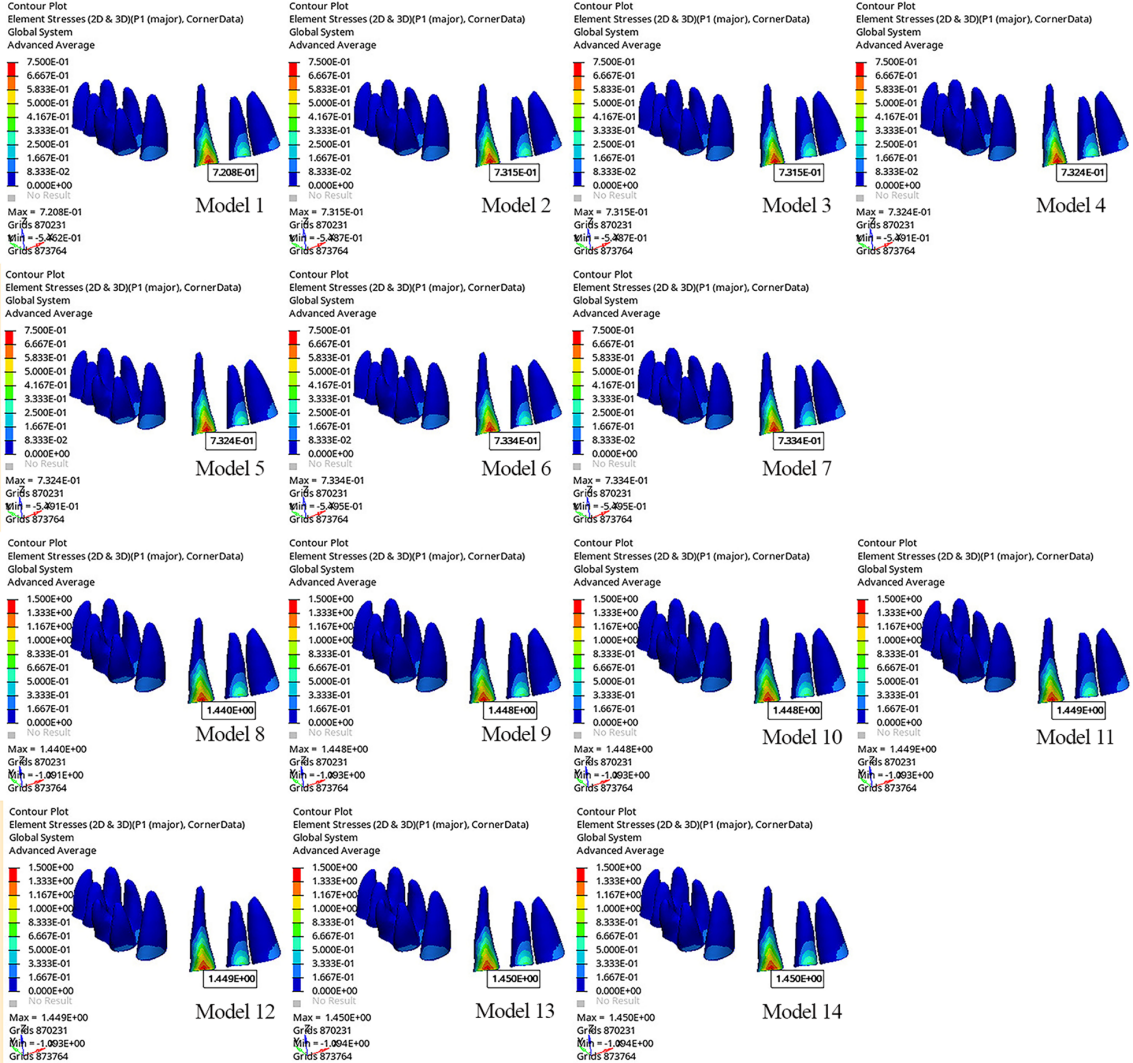
Maximum principal stress of the periodontal ligament

The greatest maximum principal stress was observed in the PDL surrounding the mesiobuccal line of the canine crown in all models (Fig. 8). The highest maximum principal stress in the PDL was observed in Models 13 and 14 (1.450 MPa), while the lowest maximum principal stress was observed in Model 1 (0.7208 MPa). The highest minimum principal stress in the PDL was observed in Models 9–14 (− 1.093 MPa), and the lowest minimum principal stress was observed in Model 1 (− 0.5462 MPa). As aligner activation and power arm length increased, the maximum principal stress in the PDL also increased (Table 7).

**Table 7:** Principal stress values of the canine tooth in the periodontal ligament

Models	Principal stress (Mpa)	
	Max	Min
Model 1	0.7208	− 0.5462
Model 2	0.7315	− 0.5487
Model 3	0.7315	− 0.5487
Model 4	0.7324	− 0.5491
Model 5	0.7324	− 0.5491
Model 6	0.7334	− 0.5495
Model 7	0.7334	− 0.5495
Model 8	1.440	− 1.091
Model 9	1.448	− 1.093
Model 10	1.448	− 1.093
Model 11	1.449	− 1.093
Model 12	1.449	− 1.093
Model 13	1.450	− 1.093
Model 14	1.450	− 1.093

*Mpa* megapascal; *Max*: maximum; *Min* minimum

When models were compared in terms of power arm material, i.e., SS or FRC, similar canine and aligner displacement was observed in models with the same power arm length and aligner activation on the x-, y-, and z-axes (Figs. 2, 3, and 4; Table 4).

The von Mises stress of SS power arms was higher than that of FRC power arms of the same length in all models. The stress in the FRC power arms was closer to the gingival tissue. The lowest von Mises stress was observed in Model 3, at 87.06 MPa (Fig. 7; Table 6).

When models with the same degree of aligner activation and same power arm length were evaluated among themselves, the effect of using SS or FRC as the power arm material on the maximum principal stress was the same; as aligner activation increased, the minimum principal stress increased. Although the minimum principal stress increased with increasing power arm length in models with 0.1 mm of aligner activation, the same values were observed for different power arm lengths and materials in models with 0.2 mm of aligner activation.

## Discussion

With the use of auxiliary equipment (buttons, miniscrews, power arms, attachments, and elastics), challenging orthodontic cases have become treatable with CAs [5, 9, 30, 41, 42].

The effects of aligner activation and power arm length on canine and aligner displacement, as well as the von Mises stress of the power arm and the principal stress of the PDL, were evaluated in this study via FEA. In addition, the effect of SS and FRC as the power arm material was also evaluated under conditions of different aligner activations and power arm lengths. To our knowledge, there have been no studies evaluating the effectiveness of different aligner activations and power arm lengths on canine distalization by CA treatment. Furthermore, there have been no studies comparing CAs with different power arm materials.

Under 0.2 mm of aligner activation, increasing the length of the power arm resulted in increased canine displacement along the x-, y-, and z-axes, increased von Mises stress on the aligner and power arm, and increased maximum principal stress on the PDL.

Robert et al. described force should be applied to teeth by between 0.15 and 0.25 mm of activation during CA treatment [43]. Lil et al. described that optimal stress and strain values can be achieved with optimal aligner activation, which is in the range of 0.07–0.24 mm [44]. Barbagallo et al. reported that 0.5 mm of aligner activation causes sudden deformation of the aligner, resulting in a decrease in the force applied by the aligner to the tooth [45]. In the present study, activation values similar (0.1 and 0.2 mm) to those exerting the optimal force were applied in FEA.

The length of the power arm [13, 46], the distance between the force and the center of resistance [46], and the direction of the applied force vector [47] affect tooth movement and stress distribution. In the present study, under 0.2 mm of CA activation, increasing the length of the power arm increased the palatal tipping of the canine crown, displacement of the CA, and the stress of the canine PDL. However, distal tipping of the canine crown was reduced using longer power arms. Declerk et al. reported that placing the power arm at the level of the center of resistance of the canine created more parallel movement [48]. Although the 13-mm power arm was situated closest to the center of resistance, the greatest parallel movement was achieved with the 14-mm power arm in this study. This is because the force was not applied only through the power arm. According to the models, force was provided by both aligner activation and the power arm. Greater proximity of the resultant force (from the power arm and aligner activation) to the center of resistance was provided by increasing the length of the power arm. Thus, compared with no power arm, power arms of increasing length caused more parallel movement.

Xu et al. evaluated the effect of auxiliary types of equipment (attachments and power arms) on canine distalization by CA treatment. Distal tipping of the canine crown was more common in the group with no attachments and in the group with vertical rectangular attachments than in the group with a power arm [30]. In the present study, only a vertical rectangular attachment was used in Models 1 and 8, while a power arm and vertical rectangular attachment were used in the other models. Models 1 and 8 demonstrated the most distal tipping of the canine crown.

The observed rotation of the canine in the distopalatal direction is similar to the findings reported by Shpack et al. [49]. This rotation occurs because the forces from the CA and the power arm pass through the labial aspect of the center of resistance of the tooth on the transverse plane. Distopalatal rotation was also observed in the extrusion of the canine crown. Greater canine crown extrusion was observed in models with 0.2 mm of aligner activation than in models with 0.1 mm of aligner activation. After classifying the models according to aligner activation (0.1 mm or 0.2 mm) and comparing models of the same class among themselves—although extra force was applied (4 oz) in models with a power arm—there was less distal tipping and extrusion than in models with only aligner activation. When models with only a power arm (12, 13, and 14 mm) were compared among themselves—although the distal tipping decreased as the length of the power arm increased—greater extrusion was observed because the increase in the palatal tipping of the canine crown was greater than the decrease in the distal tipping.

Cortona et al. reported that the region of deformation in the CA was usually in the region of tooth movement [50]. The greatest displacement of the CA was observed in the distobuccal region of the canine. Von Mises stress occurred mostly in the aligner at the distal marginal region of the canine tooth in all models, similar to the findings reported by Jing et al. [51]. In addition, the lower principal stress in the PDL in Models 1 and 8 than in the other models was due to the application of force through aligner activation only.

The displacement of the canine along the x-, y-, and z-axes, displacement of the CA, and stress on the PDL were similar for power arms made of SS and FRC, but less von Mises stress was observed in FRC power arms than in SS power arms.

The fiber type, ratio, and distribution in the composite matrix structure affect the mechanical properties of FRCs, such as the fracture toughness, compressive strength, load-bearing capacity [52], flexural strength [53], fatigue resistance [54], and fracture strength [55]. Sfondrini et al. [56] compared the flexural strength of conventional and nanofilled FRCs and found that nanofilled FRCs showed significantly higher loading strengths than conventional FRCs. In addition, Scribante et al. [57] compared the use of FRC splints and multistranded wires as retainers and found no differences in loading capacity. Therefore, it can be assumed that FRCs will be used more in dentistry in the future; FRCs could even replace metals in some orthodontic applications [58], including space protection, posttraumatic splinting, passive tooth movement (bonded canine-to-canine retainers or bonded bridges to replace missing teeth), and active tooth movement (adjuncts for active tooth movement instead of conventional SS appliances) [58]. The fiber material in FRCs can be carbon, polyethylene, glass, or polyamide [53]. In the present study, fiber composite material was preferred because of its good mechanical properties, including its high tensile strength [21] and low elastic modulus [32], as well as its inertness and biocompatibility. In addition, it exhibits low thermal and electrical conductivity, low density, high corrosion resistance, and strong bonding with resin [21, 22, 59]. In this study, the use of power arms made of FRC in CA treatment was possible because the FRC power arms resulted in similar tooth displacement as SS power arms with less stress on the power arm.

### Limitations

In this study, all models were established with homogeneous, isotropic and linear elastic materials, contrary to in vivo conditions. Therefore, a 100% accurate simulation of the in vivo environment could not be created. However, the results of FEA studies are similar to those of in vivo studies [60, 61]. Second, intraoral conditions (temperature, humidity) were not included in the FEA. Evaluation of the intraoral properties of FRC materials in future studies is recommended.

## Conclusion

The application of increased aligner activation and additional force with a power arm increased the displacement of the canine and aligner. Using a power arm in canine distalization decreased distal tipping and increased palatal tipping. A power arm may be used to reduce mesiodistal tipping in canine distalization via CA treatment. However, care should be taken in terms of palatal tipping of the canine crown. The von Mises stress increased as the length of the power arm increased. The application of increased aligner activation and additional force with a power arm also increased the principal stress in the canine PDL.

The movement of the canine on all planes (x, y, and z) and the deformation of the aligner were similar for both SS and FRC power arms. Less von Mises stress was observed in FRC than SS power arms. The principal stress of the PDL was the same for both SS and FRC power arms. FRC may be used as an alternative to SS as a power arm material. We therefore recommend that in vivo studies on the use of FRC should be conducted before clinical application.

## Data Availability

Not applicable.

## Abbreviations

SS: Stainless steel
FRC: Fiber-reinforced composite
CA: Clear aligner
PDL: Periodontal ligament
FEA: Finite element analysis
FE: Finite element
